# Dragon Fruit Allergy With Cross‐Reactivity to *Salsola kali* and Peach

**DOI:** 10.1155/crii/5321048

**Published:** 2026-06-22

**Authors:** Lucía Ferrer Clavería, Verónica Perciun, María Teresa Sobrevía Elfau, Beatriz Rojas-Hijazo, José Luis Cubero Saldaña, M. Ángeles López-Matas

**Affiliations:** ^1^ Allergology Service, Clinical University Hospital Lozano Blesa, Zaragoza, Spain, hcuz.es; ^2^ R&D Allergy and Immunology, LETI Pharma SLU, Madrid, Spain

## Abstract

Dragon fruit, also known as pitahaya, has become increasingly popular in Europe and the United States owing to its nutritional value and potential health‐promoting properties. This study describes a patient with anaphylaxis after the ingestion of dragon fruit. We present the case of a patient who experienced a systemic reaction requiring hospitalization 1 h after consuming dragon fruit. His medical history includes allergic rhinitis to *Salsola kali* pollen. The diagnostic worked‐up included prick‐by‐prick testing with fruits, skin prick testing with aeroallergens, and measurement of specific IgE using ImmunoCAP and ISAC. These tests confirmed sensitization to dragon fruit, several pollens including *S. kali*, and peach allergens. A protein extract was prepared from dragon fruit pulp. In the protein profile, the most intense bands were observed at 9, 26, and 54 kDa. The patient’s IgE recognized multiple bands ranging from 16 to >100 kDa. Immunoblot inhibition demonstrated cross‐reactivity: *S. kali* extract almost completely inhibited IgE recognition of dragon fruit proteins, leaving only faint recognition of a 54‐kDa band (identified as catalase), while peach peel extract completely inhibited IgE binding to the dragon fruit extract. In conclusion, sensitization to dragon fruit in this patient was probably due to cross‐reactivity with *S. kali* and peach allergens. This case highlights the potential risks associated with the introduction of novel foods into the diet, particularly in patients previously sensitized to pollen or other plant‐derived foods.

## 1. Introduction

Dragon fruit, also known as pitahaya, belongs to the Cactaceae family and is the fruit of cactus species of the genus *Hylocereus* (recently reclassified as *Selenicereus*). The most commonly consumed species are *Selenicereus undatus*, *Selenicereus ocamponis*, and *Selenicereus megalanthus*. This exotic fruit is native to Mexico and South America and is also cultivated in Southeast Asian countries. Dragon fruit is characterized by its striking appearance and has become popular in Europe and the United States owing to its high nutritional value and potential health‐promoting properties. It can also be used for juice production, and its agricultural byproducts are used to flavor and improve other food products [1].

## 2. Case Report

We present the case of a 26‐year‐old man who experienced a systemic reaction (generalized pruritus, hives, facial, lip, and glottic edema with dyspnea and an oxygen saturation of 92%) requiring emergency treatment with methylprednisolone (60 mg) and dexchlorpheniramine and hospitalization for 24 h. Symptoms developed approximately 1 h after ingestion of fresh dragon fruit. Acute tryptase levels were not measured. The patient has taken ibuprofen 600 mg and amoxicillin 500 mg, approximately 3 h before symptom onset; however, he continued both medications for four additional days without adverse reactions and subsequently tolerated them without incident. He reported no previous adverse reactions to other fruits but avoided further consumption of dragon fruit after this episode.

His personal history includes allergic rhinitis to *Salsola kali* pollen, which was treated with immunotherapy to *S. kali* from 2016 to 2018.

Skin prick test (SPT) with common aeroallergens were positive not only for *S. kali* (12 mm), but for other pollens such as grasses (10 mm), *Olea europaea* (10 mm), *Parietaria judaica* (10 mm), and *Platanus acerifolia* (9 mm). SPT with panallergens, including lipid transfer proteins (LTPs) and profilin was negative. The patient reported mild seasonal pollen‐related symptoms during the summer months, coinciding with *S. kali* and *P. judaica* pollination, and did not require treatment by the moment.

Food allergy was studied using the prick‐by‐prick technique with dragon fruit, locally relevant fruits (peach and cherry) and tropical fruits (kiwi and banana). The patient was positive only to dragon fruit (wheal, 7 mm; erythema, 9 mm) and peach (wheal, 6 mm; erythema, 8 mm). Notably, the patient tolerated peach without problems. Five healthy control subjects did not react to dragon fruit with prick‐by‐prick testing. Confirmation of the diagnosis with an oral food challenge (OFC) was considered clinically inadvisable due to the high risk of inducing a systemic reaction.

In vitro ImmunoCAP showed a total IgE of 375 kU/L. Positive specific IgE values were detected for peach (1.12 kU_A_/L), Pru p 1 (0.38 kU_A_/L), and Pru p 3 (3.56 kU_A_/L). ISAC microarray results were positive for most grass allergens, Cup a 1, Ole e 1, Par j 2, Sal k 1, polcalcin, mites, and cat, with the highest values being recorded for Sal k 1 (76 ISU‐E) and the LTP Par j 2 (66 ISU‐E). All values are included in Table 1. Baseline tryptase was 4.86 µg/L.

**Table 1:** Positive allergens by ImmunoCAP and ISAC.

Allergens	Biochemical name	ImmunoCAP sIgE (kU_A_/L)	ISAC sIgE (ISU)
Food allergens			
Peach	N/A	1.12	N/A
rPru p 1	Pathogenesis‐related protein, PR‐10	0.38	0.6
rPru p 3	Nonspecific lipid transfer protein, nsLTP	3.56	0.5
rPru p 4	Profilin	<0.1	<0.1
rPru p 7	Gibberellin‐regulated protein	<0.1	N/A
Ara h 9	nsLTP	N/D	0.9
Cor a 8	nsLTP	N/D	0.4
Jug r 3	nsLTP	N/D	0.4
Aeroallergens			
Art v 3	nsLTP	N/D	0.5
Bet v 4	Polcalcin	N/D	44
Pla a 3	nsLTP	N/D	6.3
Cup a 1	Pectate lyase	N/D	0.6
Ole e 1	Common olive group 1	N/D	29
Cyn d 1	Beta‐expansin	N/D	4.1
Phl p 1	Beta‐expansin	N/D	22
Phl p 4	Berberine bridge enzyme	N/D	8.1
Phl p 5	Grass pollen allergen group 5/6	N/D	7.6
Phl p 6	Grass pollen allergen group 5/6	N/D	5.8
Phl p 7	Polcalcin	N/D	39
Par j 2	nsLTP	N/D	66
Sal k 1	Pectin methylesterase	N/D	76
Fel d 4	Lipocalin	N/D	1.4
Der f 2	NPC2 family	N/D	7.9
Der p 2	NPC2 family	N/D	13
CCDs (MUXF3)	N/A	N/D	2.2

Abbreviations: N/D, not determined by ImmunoCAP; N/A, not applicable.

Dragon fruit was purchased at a local market, and the pulp was used to produce the protein extract. Briefly, dragon fruit pulp was homogenized and extracted for 30 min in phosphate‐buffered saline (PBS)/polyvinylpolypyrrolidone buffer under continuous magnetic stirring at 4°C. The extract was then centrifuged for 30 min at 5300 × *g*, and the supernatant was collected, dialyzed, filtered, frozen, and freeze‐dried. The protein concentration was determined following the Bradford method, which yielded 16.1 μg of protein/mg of lyophilized extract. *S. kali* pollen and peach peel extracts were independently prepared under Good Manufacturing Practice Guidelines (LETI Pharma, SLU, Madrid, Spain). Briefly, defatted pollen of *S. kali* (Iberpolen, Spain) was extracted in 0.01 mol/L PBS/0.15 mol/L NaCl (1:20 w/v), followed by centrifugation, filtration, dialysis, and lyophilization. Peach peel was prepared in the same way as described for dragon fruit.

**Figure 1:**
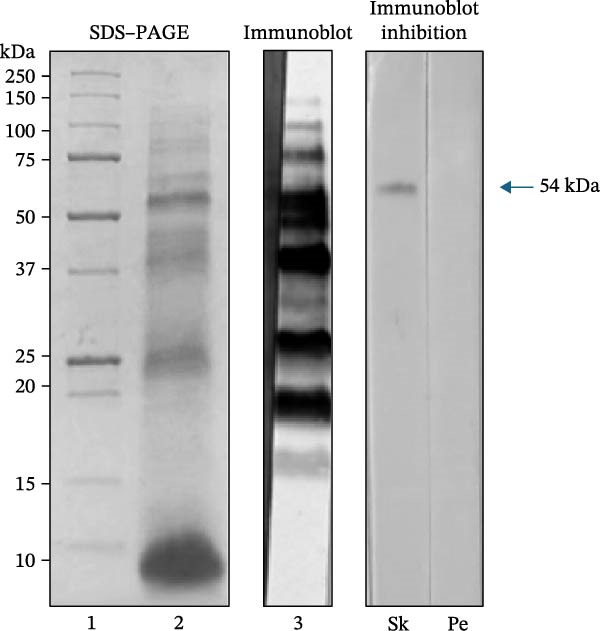
Protein profile, allergenic profile, and cross‐reactivity of dragon fruit. Lane 1: Precision Plus molecular weight marker (BioRad). Protein (SDS–PAGE, lane 2) and allergenic (Immunoblot, lane 3) profile of dragon fruit. Cross‐reactivity (immunoblot inhibition); the extract used as inhibitor is shown under the image; Sk‐inhibition with 75 µg protein of *S. kali* protein; Pe‐inhibition with 75 µg protein of peach peel extract. A total of 15 µg of dragon fruit extract protein was loaded in the solid phase in all lanes. The band of 54 kDa that was only partially inhibited with *S. kali* extract was marked with an arrow. Patient serum at a 1/2 dilution (immunoblot) and 1/4 dilution (immunoblot inhibition).

Fifteen micrograms of dragon fruit protein extract were loaded into a TGX gel (BioRad, Hercules, CA, USA) to study the protein profile by SDS–PAGE. The most intense bands were those at 9, 26, and 54 kDa (Figure 1, lane 2).

Protein recognition by IgE was studied using immunoblot. Proteins separated by SDS–PAGE were transferred to polyvinylidene difluoride (PVDF) membranes (BioRad) and incubated with the patient’s serum or with sera from 3 nonatopic controls at a 1/2 dilution. A negative control without serum was also included to discard unspecific recognition of IgE. HRP‐conjugated mouse anti‐human IgE (Southern Biotech, Birmingham, AL, USA) was used as a secondary antibody, and Amersham ECL Prime Western Blotting Detection Reagent (Cytiva, Marlborough, MA, USA) was used to detect allergenic proteins by chemiluminescence. The patient’s IgE recognized several bands, ranging from 16 to >100 kDa, the most intense being those at approximately 18, 27, 37, 47, and 54 kDa (Figure 1, lane 3). No IgE binding was observed with control sera (data not shown).

Cross‐reactivity with other allergens was studied using immunoblot inhibition. Serum was pre‐incubated at a 1/4 dilution for 2 h with *S. kali* or peach peel extracts. The amount of protein from these extracts used as inhibitor (75 µg) corresponded to five times the amount of dragon fruit protein coated on the solid phase (15 µg). The *S. kali* extract almost completely inhibited recognition of dragon fruit, leaving only a slight recognition of the 54‐kDa band. The peach peel extract completely inhibited the dragon fruit extract. These results can be seen in Figure 1 (immunoblot inhibition). The 54‐kDa band was identified by mass spectrometry, corresponding to the protein W0FHL5 (Uniprot Database). This protein is a catalase, with 78.9% of identity with the allergen Mus a 7 of banana.

## 3. Discussion

Dragon fruit allergy has previously been associated with sensitization to LTP [2] and with high‐molecular‐weight proteins (between 75 and 100 kDa) [3]. In the patient assessed, the absence of an OFC limits diagnostic certainty; however, IgE mediated sensitization was clearly demonstrated by immunoblot analysis and inhibition assays.

Immunoblot‐inhibition supports cross‐reactive IgE binding between dragon fruit and both *S. kali* and peach allergens, to which the patient was also sensitized. The prevalence of *S. kali* sensitization is high in the region of Spain where the hospital is located (Zaragoza) [4], and peach allergy is also common in Mediterranean populations [5]. Given that dragon fruit and *S. kali* belong to the order Caryophyllales, they may share allergenic proteins. Complete inhibition with *S. kali* extract, except for a partially inhibited 54‐kDa band, suggests the involvement of dragon fruit allergens similar to *S. kali* allergens. The persistence of a faint 54‐kDa signal, corresponding to a catalase, after inhibition with *S. kali* may reflect that dragon fruit catalase is a highly abundant protein that is not sufficiently represented or homologous in the *S. kali* extract. To date, no protein of the catalase family has been reported as an allergen in *S. kali* pollen. The complete inhibition observed with peach peel extract suggests that the residual band may share antigenic determinants with peach components. Since catalase has been reported as an allergen in banana [6], it is plausible that it may also be present in other fruits, such as dragon fruit. To date, the WHO/IUIS database contains seven *S. kali* allergens [7], of which six (Sal k 1 [43 kDa], Sal k 2 [36 kDa], Sal k 3 [39 + 45 kDa], Sal k 4 [14 kDa], Sal k 5 [18.2 kDa], and Sal k 6 [47 kDa]) have molecular weights that are similar to those found in dragon fruit allergens. In 2022, Hao et al. [8] correlated dragon fruit allergy with previous sensitization to pollen. In the present case, dragon fruit allergy may be associated with prior sensitization to *S. kali*. Notably, the patient had been treated with AIT to *S. kali*, highlighting that pollen AIT does not necessarily confer clinical tolerance to homologous food allergens. Indeed, evidence for improvement of pollen‑related food reactions with pollen AIT remains inconsistent across trials [9].

Peach peel inhibition was complete, although the patient tolerated this fruit. This discrepancy may be explained by differences in allergen distribution between peel and pulp, as major peach allergens such as Pru p 3 are concentrated in the peel. The patient showed clear sensitization to Pru p 3 by ImmunoCAP and ISAC. The Pru p 3 value of 3.56 kUA/L on ImmunoCAP is clearly indicative of genuine LTP sensitization, and the detectable signal on ISAC (0.5 ISU‐E), although numerically low, is consistent with genuine LTP sensitization within the context of the microarray’s lower sensitivity [10]. Despite Pru p 3, other allergens present in the peach extract were able to inhibit recognition of dragon fruit allergens, as all dragon fruit allergens were inhibited with the peach extract. Therefore, other peach allergens, such as Pru p 2 or R‐mandelonitrile lyase, could be involved in the cross‐reactivity with dragon fruit detected in this patient. In this sense, Pru p 2 has been related to fruit‐pollen allergy [11].

The patient showed evidence of LTP sensitization (Pru p 3 3.56 kUA/L on ImmunoCAP; low‑level signal on ISAC), despite clinical tolerance to peach and negative SPT to LTP panallergen. This illustrates that sensitization does not necessarily imply clinical reactivity, and that extract composition (peel vs. pulp) and potential cofactors may influence reaction thresholds and inhibition patterns. Moreover, since LTP has not been described as a relevant allergen in *S. kali*, this made it difficult to establish a direct mechanistic link between *S. kali* pollen sensitization and LTP‑mediated fruit reactions in this patient. The major allergen of *S. kali*, Sal k 1 only shows cross‑reactivity to the *Salsola* genus rather than to foods; therefore, tolerance induced by pollen AIT would not be expected to extend to plant foods. The immunoblot‑inhibition pattern suggests that dragon fruit recognition is mediated by shared proteins beyond Sal k 1 and by peach components (including LTPs), underscoring that post‑AIT food risk persists.

IgE binding to cross‑reactive carbohydrate determinants (CCDs) must also be considered, as the patient showed MUXF3 reactivity on ISAC (2.2 ISU‐E). However, CCD‐specific IgE rarely correlates with clinical symptoms [12], which is not the case of the patient studied, who had anaphylaxis with dragon fruit. Cofactors (NSAIDs and amoxicillin) could have been an aggravating factor [13], and therefore, limits the ability to predict how common these kinds of reactions are in other *S. kali*‐sensitized individuals.

In conclusion, the patient’s sensitization to dragon fruit is consistent with cross‑reactivity with *S. kali* and peach allergens, and the episode occurred in the presence of potential cofactors that may lower reaction thresholds. Several explanations remain plausible in this case, including independent co‑sensitization to dragon fruit, *S. kali*, and peach; IgE binding to CCDs; or primary sensitization to one source with subsequent cross‑recognition.

This case warns of the introduction of new foods in the diet, especially in patients previously sensitized to pollen or other foods. Allergenic dragon fruit proteins and the determinants of cross‐reactivity with pollen and other fruits have yet to be characterized.

## Funding

No external funding was received.

## Consent

Written informed consent was obtained from the patient for the publication of this case report.

## Conflicts of Interest

The authors declare no conflicts of interest.

## Data Availability

The data that support the findings of this study are available from the corresponding author upon reasonable request.

## References

[1] Saimen E. , Marbawi H. , Goh L. P. W. , Sabullah M. K. , Gansau J. A. , and Jawan R. , Unleashing the Potential of Fruit Byproducts in Value-Added Foods for Sustainable Nutrition: A Review, Journal of Biochemistry, Microbiology and Biotechnology. (2025) 13, no. 1, 21–29, 10.54987/jobimb.v13i1.1071.

[2] Kleinheinz A. , Lepp U. , Hausen B. M. , Petersen A. , and Becker W.-M. , Anaphylactic Reaction to (Mixed) Fruit Juice Containing Dragon Fruit, Journal of Allergy and Clinical Immunology. (2009) 124, no. 4, 841–842, 10.1016/j.jaci.2009.05.025. 19596400

[3] Rodriguez-Jimenez B. , Dominguez-Ortega J. , Ledesma A. , Cava-Sumner B. , and Kindelan-Recarte C. , Generalized Urticaria Due to Yellow Pitahaya (*Selenicereus Megalanthus*), Journal of Investigational Allergology & Clinical Immunology. (2014) 24, no. 2, 124–125. 24834776

[4] Barber D. , de la Torre F. , and Lombardero M. , et al.Component-Resolved Diagnosis of Pollen Allergy Based on Skin Testing With Profilin, Polcalcin and Lipid Transfer Protein Pan-Allergens, Clinical & Experimental Allergy. (2009) 39, no. 11, 1764–1773, 10.1111/j.1365-2222.2009.03351.x. 19877313

[5] Barni S. , Caimmi D. , and Chiera F. , et al.Phenotypes and Endotypes of Peach Allergy: What Is New?, Nutrients. (2022) 14, no. 5, 10.3390/nu14050998, 998. 35267973 PMC8912752

[6] Nikolić J. , Nešić A. , Kull S. , Schocker F. , Jappe U. , and Gavrović-Jankulović M. , Employment of Proteomic and Immunological Based Methods for the Identification of Catalase as Novel Allergen from Banana, Journal of Proteomics. (2018) 175, 87–94, 10.1016/j.jprot.2018.01.007. 29331514

[7] World Health Organization , International Union of Immunological Societies Allergen Nomenclature Sub-committee, Allergen Nomenclature., https://www.allergen.org.

[8] Hao M. , Xijiri , Zhao Z. , and Che H. , Identification of Allergens in White- and Red-Fleshed Pitaya (*Selenicereus Undatus* and *Selenicereus Costaricensis*) Seeds Using Bottom-Up Proteomics Coupled With Immunoinformatics, Nutrients. (2022) 14, no. 9, 10.3390/nu14091962, 1962. 35565931 PMC9134757

[9] Al-Shaikhly T. , Cox A. , and Nowak-Wegrzyn A. , et al.An International Delphi Consensus on the Management of Pollen-Food Allergy Syndrome: A Work Group Report of the AAAAI Adverse Reactions to Foods Committee, The Journal of Allergy and Clinical Immunology: In Practice. (2024) 12, no. 12, 3242–3249.e1, 10.1016/j.jaip.2024.09.037. 39488768 PMC11625607

[10] Griffiths R. L. M. , El-Shanawany T. , and Jolles S. R. A. , et al.Comparison of the Performance of Skin Prick, ImmunoCAP, and ISAC Tests in the Diagnosis of Patients with Allergy, International Archives of Allergy and Immunology. (2017) 172, no. 4, 215–223, 10.1159/000464326. 28456812

[11] Palacín A. , Rivas L. A. , and Gómez-Casado C. , et al.The Involvement of Thaumatin-Like Proteins in Plant Food Cross-Reactivity: A Multicenter Study Using a Specific Protein Microarray, PLoS ONE. (2012) 7, no. 9, 10.1371/journal.pone.0044088, e44088. 22970164 PMC3436791

[12] Kamath S. D. , Bublin M. , Kitamura K. , Matsui T. , Ito K. , and Lopata A. L. , Cross-Reactive Epitopes and Their Role in Food Allergy, Journal of Allergy and Clinical Immunology. (2023) 151, no. 5, 1178–1190, 10.1016/j.jaci.2022.12.827. 36932025

[13] Cardona V. , Ansotegui I. J. , and Ebisawa M. , et al.World Allergy Organization Anaphylaxis Guidance 2020, World Allergy Organization Journal. (2020) 13, no. 10, 10.1016/j.waojou.2020.100472, 100472. 33204386 PMC7607509

